# Implications of the ACC/AHA risk score for prediction of heart failure: the Rotterdam Study

**DOI:** 10.1186/s12916-021-01916-7

**Published:** 2021-02-16

**Authors:** Banafsheh Arshi, Jan C. van den Berge, Bart van Dijk, Jaap W. Deckers, M. Arfan Ikram, Maryam Kavousi

**Affiliations:** 1 Department of Epidemiology, Erasmus MC - University Medical Center Rotterdam, Rotterdam, The Netherlands; 2 Department of Cardiology, Erasmus MC - University Medical Center Rotterdam, Rotterdam, The Netherlands

**Keywords:** Heart failure, Prediction, Primary prevention, NT-proBNP

## Abstract

**Background:**

Despite the growing burden of heart failure (HF), there have been no recommendations for use of any of the primary prevention models in the existing guidelines. HF was also not included as an outcome in the American College of Cardiology/American Heart Association (ACC/AHA) risk score.

**Methods:**

Among 2743 men and 3646 women aged ≥ 55 years, free of HF, from the population-based Rotterdam Study cohort, 4 Cox models were fitted using the predictors of the ACC/AHA, ARIC and Health-ABC risk scores. Performance of the models for 10-year HF prediction was evaluated. Afterwards, performance and net reclassification improvement (NRI) for adding NT-proBNP to the ACC/AHA model were assessed.

**Results:**

During a median follow-up of 13 years, 429 men and 489 women developed HF. The ARIC model had the highest performance [c-statistic (95% confidence interval [CI]): 0.80 (0.78; 0.83) and 0.80 (0.78; 0.83) in men and women, respectively]. The c-statistic for the ACC/AHA model was 0.76 (0.74; 0.78) in men and 0.77 (0.75; 0.80) in women. Adding NT-proBNP to the ACC/AHA model increased the c-statistic to 0.80 (0.78 to 0.83) in men and 0.81 (0.79 to 0.84) in women. Sensitivity and specificity of the ACC/AHA model did not drastically change after addition of NT-proBNP. NRI(95%CI) was − 23.8% (− 19.2%; − 28.4%) in men and − 27.6% (− 30.7%; − 24.5%) in women for events and 57.9% (54.8%; 61.0%) in men and 52.8% (50.3%; 55.5%) in women for non-events.

**Conclusions:**

Acceptable performance of the model based on risk factors included in the ACC/AHA model advocates use of this model for prediction of HF risk in primary prevention setting. Addition of NT-proBNP modestly improved the model performance but did not lead to relevant discrimination improvement in clinical risk reclassification.

**Supplementary Information:**

The online version contains supplementary material available at 10.1186/s12916-021-01916-7.

## Background

Heart failure (HF) remains a major public health problem among men and women worldwide [1, 2]. The growing morbidity and mortality of HF, along with poor quality of life and prognosis, high costs, and the challenges of treating clinically overt HF highlight the need for more efficient preventive strategies [3, 4]. To identify high risk individuals who would benefit most from early prevention, several HF risk prediction models have been developed [5, 6]. However, none of these models have been recommended for routine use in clinical practice [5].

The recent American College of Cardiology (ACC)/American Heart Association (AHA) guidelines use the pooled cohort equations (PCE) to predict 10-year risk of atherosclerotic cardiovascular disease (ASCVD). Compared to the previous guidelines, the newer guidelines have expanded the focus from coronary heart disease (CHD) only to an ASCVD outcome that additionally includes stroke [7]. Due to variability between studies in ascertainment of HF, incident HF has not been included in this newly expanded outcome. The PCE is comprised of the traditional cardiovascular risk factors which were among the ten most consistently reported predictors included in HF prediction models in a recent meta-analysis [6]. Compared to the more specific HF prediction models, risk factors included in the PCE are simple to measure and available in most clinical settings.

In this study, we assessed the performance of a model fitted based on the risk factors used in the PCE (ACC/AHA model) for 10-year HF prediction among men and women from the large prospective population-based Rotterdam Study. We also compared the performance of this model for HF prediction with the performance of models based on risk factors included in the two risk scores that have been specifically developed and validated to predict HF in the general population; namely the Atherosclerosis Risk in Communities (ARIC) and the Health Aging and Body Composition (Health ABC) HF risk scores [8, 9]. Furthermore, we investigated whether addition of NT-proBNP, to the ACC/AHA risk score improved HF risk prediction.

## Methods

### Study sample

This project was carried out within the framework of the Rotterdam Study, a prospective population-based study among subjects 45 years and older in Rotterdam, the Netherlands. The baseline examination of the Rotterdam Study included 7983 individuals and was completed between 1989 and 1993 (RS-I). The cohort has been extended twice (3011 individuals, RS-II, recruited in 2000–2001 and 3932 individuals, RS-III, in 2006) to include participants who were 45 years or older or had moved to the study area. Rotterdam Study participants have been followed up ever since and the examinations have been repeated every 3–4 years. The overall response for all three study cycles at entry was 72.0% (14,926 of 20,744). The rationale and design of the study have been previously described [10]. The Rotterdam Study has been approved by the Medical Ethics Committee of the Erasmus MC (registration number MEC 02.1015) and by the Dutch Ministry of Health, Welfare, and Sport (Population Screening Act WBO, license number 1071272-159521-PG). The Rotterdam Study has been entered into the Netherlands National Trial Register (NTR; www.trialregister.nl) and into the WHO International Clinical Trials Registry Platform (ICTRP; www.who.int/ictrp/network/primary/en/) under shared catalog number NTR6831. All participants provided written informed consent to participate in the study and to have their information obtained information from their treating physicians.

**Fig. 1:**
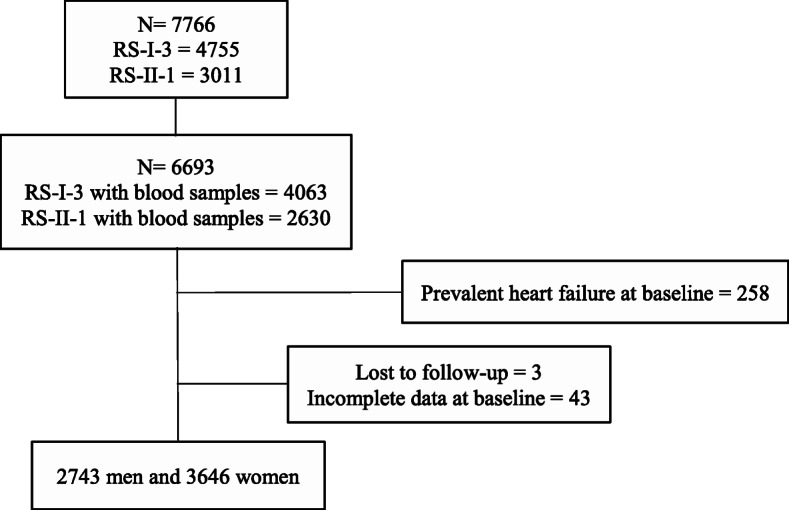
Flowchart of the included study participants

The present study used data from the third examination of the original cohort (RS-I-3, 1997-1999, *n* = 4755) and the first examination of the extended cohort (RS-II-1, *n* = 3011) had blood samples (RS-I-3, *n* = 4063 and RS-II-1, *n* = 2630). We excluded participants with a history of HF at baseline (*n* = 258), those with incomplete data at baseline or lost to follow-up (*n* = 46). After exclusions, 6389 participants (2743 men, 3646 women) were included in the study (Fig. 1).

### Predictors of HF

Body mass index (BMI) was calculated based on weight in kilograms divided by height in meters squared. Blood pressure was measured on the right arm using a random-zero sphygmomanometer at sitting position. Two measurements were performed and the average of the two was used in the analyses. Antihypertensive treatment for hypertension, use of lipid lowering medication, history of diabetes mellitus, and history of CHD were based on clinical information obtained from general practitioners and letters or discharge reports from medical specialists [11]. Information on smoking behavior was acquired from questionnaires. For the ACC/AHA model, participants were classified as current smokers versus former or never smokers. For the Health ABC and ARIC models, smoking status was classified as current, former, and never. Fasting serum glucose levels were determined using the glucose hexokinase method and serum total and high-density lipoprotein (HDL) cholesterol were measured using an automatic enzymatic procedure (Hitachi 911, Roche CHOD PAP). Serum creatinine levels were measured using an enzymatic assay (Roche Diagnostics, Mannheim, Germany) which was calibrated by isotope dilution mass spectrometry. Serum NT-proBNP was measured using a commercially available electrochemiluminescence immunoassay (Elecsys proBNP, F Hoffman-La Roche Ltd.) on an Elecsys 2010 analyzer [12]. Left ventricular hypertrophy (LVH) was diagnosed based on Sokolov-Lyon criteria by the Modular ECG Analysis System program with an algorithm taking into account QRS voltages with an age-dependent correction and repolarization [13]. Presence of atrial fibrillation (AF) was based on the clinical and ECG evidence from medical records [11].

### HF assessment

Ascertainment of HF for the Rotterdam Study has been previously described [11]. Information on prevalent HF cases at entry were obtained from a database containing hospital discharge diagnoses from all hospitals in Rotterdam at entry [11, 14]. During follow-up, diagnosis of incident HF was also based on clinical information systematically collected from the general practitioner medical records and verified hospital discharge diagnoses collected from all hospitals in Rotterdam. Based on the criteria of the European Society of Cardiology (ESC), the diagnosis of definite HF was based on the presence of two signs or symptoms suggestive of HF, established by objective evidence of cardiac dysfunction, confirmed by a medical specialist [15]. HF was classified as probable if at least two typical symptoms of HF were present and at least one of the following: history of CVD (MI, valvular heart disease, hypertension), response to treatment for HF, or objective evidence of cardiac dysfunction, while symptoms could not be attributed to another disease. In accordance with the ESC guidelines, only definite and probable cases were used in the Rotterdam Study definition [15].

The incident date for HF was defined as the date of the first occurrence of symptoms suggestive of HF from the medical records or the day of receipt of a first prescription for a loop diuretic or an angiotensin-converting enzyme inhibitor, whichever one preceded [11].

### Statistical analysis

Baseline characteristics of men and women were presented as mean [standard deviation (SD)] for normally distributed data and as median [interquartile range (IQR)] for skewed data and were compared using the Student *t* tests for continuous variables and *χ*^2^ tests for categorical data. Logarithmic transformation was made on NT-proBNP to account for its skewed distribution. We used multiple imputation for missing values on covariates (All missing < 5%) [16]. Parameter estimates were obtained by pooling 5 imputed datasets using Rubin rules [16].

Three different Cox proportional hazards models were developed by refitting risk factors from the PCE risk score (ACC/AHA model), the ARIC HF risk score (ARIC model) and the Health ABC HF risk score (Health ABC model). Although these models were refitted, for simplicity, we call them ACC/AHA, ARIC, and Health ABC models. Ten-year HF risk was estimated per model. Predictors included in the ACC/AHA model were age, total and HDL cholesterol, systolic blood pressure, antihypertensive treatment, current smoking, and history of diabetes. The ARIC model included age, heart rate, systolic blood pressure, antihypertensive treatment, history of diabetes, history of CHD, smoking status (current, former, never), BMI and NT-proBNP. The Health ABC model included age, history of CHD, LVH, systolic blood pressure, heart rate, smoking status (current, former, never), glucose, and creatinine. Due to unavailability, albumin measurement was left out of the Health ABC model. In addition, a fourth model was built that additionally included NT-proBNP in the ACC/AHA model as a predictor (ACC/AHA + NT-proBNP model). All models were separately developed for men and women.

For each model, a full model including interaction terms between age and NT-proBNP and between SBP and antihypertensive medication use (if applicable) and natural splines with 2 knots for age and NT-proBNP (if applicable) was first specified, forcing on the variables of the respective risk scores. Schoenfeld’s test of residuals using the Kaplan-Meier estimate of the survival function was used to check the proportionality of the regressions. Then, backward selection was performed using log likelihood ratio to compare all these nested models. A *P* value of 0.2 was considered for inclusion of nonlinear and interaction terms in multivariable models.

To compare the models, Akaike information criterion (AIC) was used. Calibration of the models was graphically evaluated by creating model-based risk plots and was further assessed with the Greenwood-D’Agostino-Nam test [17]. The discriminative performance of the fitted models was assessed by calculating the modified c-statistic by using the technique of inverse probability of censoring weighting (IPCW) for censored data [18].

To evaluate the implication of NT-proBNP on risk assessment, performance of the ACC/AHA and the ACC/AHA + NT-proBNP models for 10-year HF prediction were compared. We assessed the performance of the two models by calculating the time-dependent sensitivity, specificity, positive and negative predicted values for survival data. To do this, risk cutoffs introduced by the ACC/AHA guideline for ASCVD (5%, 7.5%, and 20%) were used [19]. We also calculated continuous and categorical NRIs. Reclassification tables were constructed to investigate the number of individuals with and without the HF event, reclassified to a higher or lower category of 10-year risk for HF. Risk categories were defined using the same cutoffs [low-risk (< 5%), borderline risk (≥ 5% and < 7.5%), intermediate risk (≥ 7.5% and < 20%), and high risk (≥ 20%)].

The original ACC/AHA model was developed for ASCVD risk calculation among asymptomatic individuals. Therefore, as a sensitivity analysis, the performance of the ACC/AHA model was also evaluated after addition of CHD history to the model. Furthermore, all analyses were once repeated in a sample with further exclusions for prevalent CHD and AF and use of lipid lowering medication based on the ACC/AHA guidelines.

All analyses were performed using R version 3.6.1 (Packages: mice, rms, survC1, timeROC, ggplot2).

## Results

Mean (SD) age was 68.0 (7.78) years in men and 69.2 (8.58) years in women (Table 1). Mean BMI (kg/m^2^) was slightly higher in women [26.5 (3.27) in men versus 27.3 (4.39) in women]. Glucose and creatinine levels were higher in men [6.09 (1.70) mmol/l in men and 5.87 (1.47) mmol/l in women for glucose and 89.0 (18.2) mmol/l in men and 70.8 (13.5) mmol/l in women for creatinine]. However, total and HDL cholesterol levels were higher in women. Thirty percent of men and 35% of women used antihypertensives while 14% and 12% took lipid lowering medications, respectively. More men (13.8%) had a history of CHD than women (3.3%). Likewise, more men had diabetes (14.7% compared to 11.6% in women). Median NT-proBNP levels were higher in women [median (IQR): 8.19 (13.4) in men and 10.8 (13.2) in women]. Data on covariates were missing for less than 5% in men and women.

**Table 1:** Characteristics of the study population

Clinical features	Men (*N* = 2743)	Women (*N* = 3646)	*P* value*
Age, years	68.0 (7.78)	69.2 (8.58)	< 0.001
BMI, kg/m^2^	26.5 (3.27)	27.3 (4.39)	< 0.001
Systolic blood pressure, mmHg	142 (20.8)	143 (21.5)	0.350
Hear rate, bpm	69.4 (11.9)	71.7 (10.9)	0.032
Total cholesterol, mmol/l	5.54 (0.95)	6.01 (0.95)	< 0.001
HDL, mmol/l	1.24 (0.32)	1.50 (0.40)	< 0.001
Antihypertensive use, *N* (%)	856 (32.7)	1220 (35.3)	< 0.001
Lipid lowering medication, *N* (%)	364 (13.8)	420 (12.0)	< 0.001
Creatinine, mmol/l^†^	89.0 (18.2)	70.8 (13.5)	< 0.001
Glucose, mmol/l	6.09 (1.70)	5.87 (1.47)	< 0.001
LVH, *N* (%)	176 (7.30)	119 (3.80)	0.034
NT-proBNP, pmol/l^†^	8.19 (13.4)	10.8 (13.2)	< 0.001
Prevalent CHD, *N* (%)	370 (13.8)	118 (3.30)	< 0.001
Prevalent diabetes, *N* (%)	403 (14.7)	423 (11.6)	0.009
Smoking, *N* (%)			< 0.001
Current	652 (25.3)	614 (17.2)	–
Past	1604 (62.2)	1315 (36.8)	–
Never	322 (12.5)	1648 (46.1)	–

Data are mean (standard deviation (SD)) for continuous variables, ^†^median (interquartile range (IQR)) for skewed variables, and number (percentage) for categorical variables from the original data

Proportion of missing: Among men: SBP: 0.18%, heart rate: 0.55%, total cholesterol, BMI and glucose: each 0.9%, HDL: 1.7%, creatinine: 1.6%, NT-proBNP: 1.6%, antihypertensive use: 2%, smoking: 2.7%, LVH: 2.9%

Among women: SBP: 0.86%, heart rate: 1.43%, total cholesterol, glucose: 1.6%, antihypertensive use: 1.8%, smoking: 1.9%, BMI: 2.1%, creatinine: 1.8%, NT-proBNP: 2%, HDL: 2.6%, LVH: 2.6%

*BMI* body mass index, *CHD* coronary heart disease, *HDL* high-density lipoprotein, *LVH* left ventricular hypertrophy

*P value for differences in characteristics between men and women

During a median follow-up of 13 years, 429 and 489 incident cases of HF were identified in men and women, respectively (incident rate: 14.5 per 1000 person-years in men and 11.4 per 1000 person-years in women). Supplementary Table 1 (Additional file 1) details the multivariable-adjusted hazard ratios (HRs) and 95% confidence intervals (95% CIs) for 10-year incident HF for the ACC/AHA, the ARIC, the Health ABC and the ACC/AHA + NT-proBNP models in men and women.

Comparing the models, the Health ABC model had the lowest AIC in men and women (5651.2 and 6656.2, respectively) with 11 degrees of freedom (see Supplementary Table 2, Additional file 1). The overall fit of the ARIC model was 5953.2 in men 6908.0 in women with 9 degrees of freedom. The AIC of the ACC/AHA model was 6213.4 in men and 7503.2 in women. The AIC of the ACC/AHA model improved substantially (*P* for the log-likelihood ratio test < 0.001) after adding NT-proBNP (5970.9 in men and 7179.4 in women). Calibration plots of observed and predicted risks were reasonable (see Supplementary Figure 1, Additional file 1). The Greenwood-D’Agostino-Nam test also indicated that all models were well-calibrated (All *P* > 0.20).

**Fig. 2:**
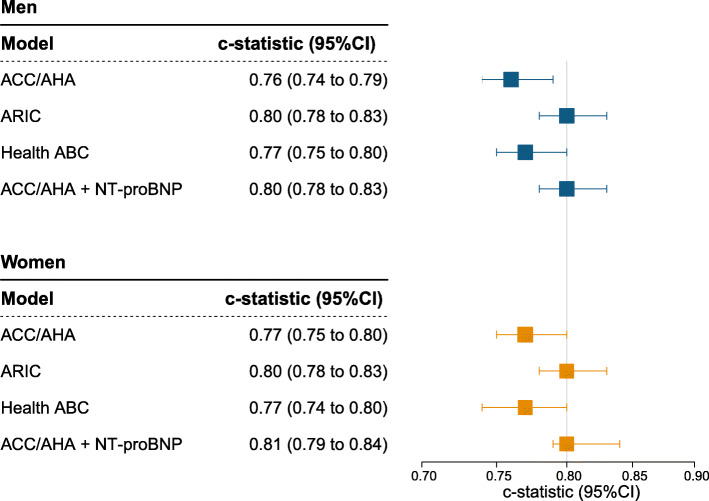
Discriminative performance of models for 10-year heart failure prediction

Figure 2 shows the discriminative performance of each model in men and women. The ARIC model had the highest discriminative ability in men and women [c-statistic (95% CI): 0.80 (0.78 to 0.83) and 0.80 (0.78 to 0.83), respectively]. The c-statistic for the ACC/AHA model was 0.76 (0.74 to 0.78) in men and 0.77 (0.75 to 0.80) in women. By adding NT-proBNP to the ACC/AHA model, the c-statistic increased to 0.80 (0.78 to 0.83) in men and 0.81 (0.79 to 0.84) in women.

**Fig. 3:**
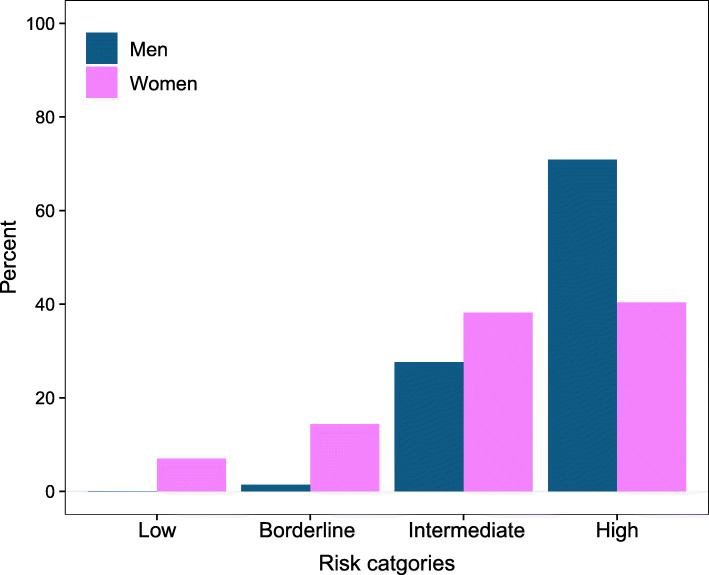
Observed risk categories based on the ACC/AHA model in men and women. Risk categories are low-risk (< 5%), borderline risk (≥ 5% and < 7.5%), intermediate risk (≥ 7.5% and < 20%), and high risk (≥ 20%)]

Using cutoffs introduced by the recent ACC/AHA guidelines, the ACC/AHA model categorized 0.04% of men as low risk, 1.5% as borderline, 27.6% as intermediate, and 70.9% as high risk (Fig. 3). Among women, the ACC/AHA model allocated 7% as low risk, 14.4% as borderline, 38.2% as intermediate, and 40.4% as high risk. Continuous NRI (95% CI) after adding NT-proBNP to the ACC/AHA model was 0.08 (− 0.08 to 0.16) in men and 0.12 (0.02 to 0.22) in women. As for categorical NRI, event NRI (95% CI) was − 23.8% (− 28.4% to − 19.2%) and non-event NRI (95% CI) was 57.9% (54.8% to 61.0%) for men. Among women, event and non-event NRI (95% CI) were − 27.6% (− 30.7% to − 24.5%) and 52.8% (50.3% to 55.5%), respectively (see Supplementary Table 3, Additional file 1). Reclassification of individuals with and without the HF event to higher or lower risk categories is depicted in (see Supplementary Figure 2, Additional file 1).

Overall the sensitivity of the ACC/AHA + NT-proBNP model was higher using different cutoffs in men and women. Specificity and predictive discrimination values were similar for both models at the 5 and 7.5% risk thresholds (Table 2). As expected, the sensitivity declined and the specificity increased for both models when risk threshold increased from 5 to 7.5% and to 20%. Using a cutoff of 20%, the ACC/AHA model correctly classified 9% of men and women who developed HF during follow-up at high risk (sensitivity). Also, 99% of men and women who remained event free during follow-up were correctly classified at low risk (specificity) by the ACC/AHA model. But, for the ACC/AHA + NT-proBNP model, the sensitivity and specificity were 16% and 99% in men, and 21% and 99% in women. From men and women categorized as ≥ 20% risk by the ACC/AHA model 88% and 57% developed HF during follow-up (positive predicted value), whereas from those categorized at low-risk group 89% of men and 92% of women remained event free during the follow-up (negative predicted value). For the ACC/AHA + NT-proBNP model, positive and negative predicted values were 84% and 90% in men, respectively. In women, the ACC/AHA + NT-proBNP model, positive and negative predicted values were 69% and 93%, respectively. Results for the analyses using the thresholds of 5%, 7.5%, and 20% 10-year HF risk are shown in Table 2.

**Table 2:** Comparison of the performance of the ACC/AHA model and ACC/AHA model with addition of NT-proBNP

	Risk thresholds		
	5%	7.5%	20%
**Men**			
Sensitivity (95% CI)			
ACC/AHA	80% (78% to 82%)	50% (47% to 53%)	9% (8% to 11%)
ACC/AHA + NT-proBNP	83% (81% to 85%)	58% (55% to 61%)	16% (14% to 18%)
Specificity (95% CI)			
ACC/AHA	70% (69% to 71%)	90% (89% to 91%)	99% (99% to 100%)
ACC/AHA + NT-proBNP	72% (71% to 73%)	90% (89% to 91%)	99% (99% to 100%)
Positive predicted value (95% CI)			
ACC/AHA	27% (25% to 28%)	40% (37% to 43%)	88% (80% to 96%)
ACC/AHA + NT-proBNP	29% (27% to 30%)	44% (41% to 46%)	84% (79% to 90%)
Negative predicted value (95% CI)			
ACC/AHA	96% (96% to 97%)	93% (92% to 93%)	89% (87% to 89%)
ACC/AHA + NT-proBNP	97% (96% to 97%)	94% (93% to 95%)	90% (89% to 90%)
**Women**			
Sensitivity (95% CI)			
ACC/AHA	81% (79% to 83%)	52% (49% to 55%)	9% (8% to 12%)
ACC/AHA + NT-proBNP	85% (83% to 87%)	63% (60% to 66%)	21% (18% to 23%)
Specificity (95% CI)			
ACC/AHA	67% (66% to 78%)	89% (88% to 90%)	99% (99% to 100%)
ACC/AHA + NT-proBNP	69% (68% to 70%)	87% (86% to 88%)	99% (99% to 100%)
Positive predicted value (95% CI)			
ACC/AHA	19% (18% to 20%)	31% (29% to 33%)	57% (48% to 67%)
ACC/AHA + NT-proBNP	21% (20% to 22%)	33% (31% to 35%)	69% (64% to 74%)
Negative predicted value (95% CI)			
ACC/AHA	97% (97% to 98%)	95% (95% to 96%)	92% (91% to 93%)
ACC/AHA + NT-proBNP	98% (98% to 99%)	96% (95% to 96%)	93% (92% to 93%)

In sensitivity analyses, prevalent CHD was added to the ACC/AHA and the ACC/AHA + NT-proBNP models. We also repeated the analyses excluding participants with AF and prevalent CHD and those using lipid lowering medication according to the ACC/AHA guidelines. The performance of the models did not change substantially (data not shown).

## Discussion

A simple model based on traditional cardiovascular risk factors included in the ACC/AHA model showed a reasonable performance in predicting 10-year HF among men and women from the population-based Rotterdam Study. The performance of the model based on ACC/AHA risk factors for HF prediction was comparable to the models based on risk factors included in the ARIC and Health ABC HF models. Adding NT-proBNP to the ACC/AHA model modestly improved model performance but did not lead to relevant clinical improvement in risk reclassification.

Compared to CHD and stroke, prediction of incident HF remains a challenge [5, 6]. In spite of the large number of risk prediction models developed for incident HF, there have been no recommendations for routine clinical use of any of the models in the existing guidelines [5]. This is while, preventive interventions significantly reduce the risk of incident HF [3]. In addition to generalizability issues and methodological heterogeneity, models specifically developed to predict HF are based on various markers which have higher technical demands and might not be available in all clinical settings [5, 8, 9, 20]. HF can have ischemic or non-ischemic origins. Although CHD and hypertension are the leading causes of HF, a high proportion of this syndrome is attributed to other cardio-metabolic risk factors [21]. Moreover, biomarkers have shown limited predictive capability for HF risk stratification and have not profited clinical decision making [22]. The ACC/AHA model for ASCVD risk assessment consists of the traditional cardiovascular risk factors that are also associated with HF [7]. In our analysis, the model based on traditional cardiovascular risk factors included in the ACC/AHA model had a performance almost similar to the Health ABC model and close to the ARIC model for HF risk assessment in general population. In a meta-analysis of HF prediction models, among the 53 potential predictors considered in 19 studies between 1990 and 2016, age, sex and systolic blood pressure were the most common selected predictors [6]. To note, the predictors in the ACC/AHA model were among the 10 most used predictors in these dedicated HF prediction models. They are also commonly used in predicting HF prognosis and other cardiovascular outcomes [23]. The acceptable performance of the model based on risk factors from the ACC/AHA algorithm for HF prediction in our study advocates implementing this model for primary HF prevention. Addition of NT-proBNP to the ACC/AHA model improved model performance in both sexes. There was overlap between men and women in the discrimination of both the ACC/AHA and the ACC/AHA + NT-proBNP models. Nevertheless, the improvement in the c-statistic after addition of NT-proBNP was slightly greater among women. To note, levels of NT-proBNP/BNP have not shown to be different between men and women with acute or chronic HF [24]. Also, women have slightly lower NT-proBNP/BNP levels in clinical setting which has been attributed to higher prevalence of HF with preserved ejection fraction among women [25]. Thus, an overlap in the performance of the model by adding these biomarkers is not far from expectation.

Studies have shown mixed results regarding the contribution of NT-proBNP/BNP to improvement of CVD risk prediction in men and women [26]. NT-proBNP has displayed no or only modest impact in increasing the discriminative ability or risk classification of CVD risk prediction models in the general population [22, 25, 27–29]. On the contrary, in high-risk individuals with previous history of CVD, higher prognostic ability has been reported [25]. It should be considered that a wide variety of cardiac and non-cardiac conditions are also associated with elevated serum levels of this biomarker [30]. Moreover, high levels of NT-proBNP are associated with creatinine level, sex, age, and inversely associated with BMI independent of ventricular function [1, 19, 31]. This is probably why its discriminative ability for detection of left ventricular systolic dysfunction has been suboptimal, limiting its utility in mass screening [32].

Using different cutoffs, addition of NT-proBNP was accompanied by slight increases in the sensitivity and specificity of the models in both sexes. Only at the 20% cutoff, the difference in the sensitivity and specificity of the two models was more evident and NT-proBNP increased the NPV and PPV of the model more evidently. Also, continuous NRI in men was smaller than women. However, using the ACC/AHA risk cutoffs, NT-proBNP mainly correctly down-classified participants without the event in both sexes and did not show large improvement in reclassifying participants with the event. In line with our study, Willeit et al. showed a strong association between NT-proBNP and the composite outcome of stroke, CHD, and HF [28]. But the increase in the c-statistic of the model after adding NT-proBNP was modest. Interestingly, they also specified that NT-proBNP improved risk prediction by appropriately down classifying the clinical risk of those without the event. Moreover, they observed similar changes using cutoffs used by different guidelines [28].

NT-proBNP/BNP is an established diagnostic and prognostic biomarker in HF patients [31]. For prediction of incident HF alone, NT-proBNP/BNP has shown to improve model performance [8, 33, 34]. However, despite the association of increasing NT-proBNP/BNP levels with substantial risk of HF, it is not a cost-effective screening tool to assess for preclinical heart failure or LV dysfunction, limiting its utility to highly selected populations [30]. In this regard, NT-proBNP testing has a clear and valuable role in the diagnosis of CHF in the emergency diagnosis of patients with dyspnea [35]. The strength of BNP is in its ability to rule out CHF in this setting. Likewise, the favorable clinical utility of adding NT-proBNP to the ACC/AHA model in risk reclassification was mainly limited to the non-events in our study. To add, BNP has shown a high NPV to rule out diastolic dysfunction or LVH [36, 37]. The ACC/AHA + NT-proBNP model also showed a high NPV for HF in our study using different risk cutoffs but the differences were more evident among those at high risk, using a cutoff of 20% which again emphasizes that NT-proBNP might be useful in prediction of HF only among high risk populations. Specifically, this utilization might be more towards ruling out HF rather than its rule-in ability [30].

Strengths of our study are use of a large sample size and detailed long follow-up data. Our HF event adjudication was robust and well-defined. Moreover, a large set of various and precisely measured variables were available for this study. There are also limitations. The Rotterdam Study population is mainly white and 45 years of age and older, compared with the ARIC and the PCE study populations. Mean age of the study population in the ARIC study [54.1(6.0) years] was younger than our total population [69.7 (8.27)] while mean age in the health ABC study [73.6 (2.9)] was somewhat closer to our study population, but with less variability. As the strongest predictor of HF, differences in age could explain to some extent the differences in the performance of the models. Hence, we refitted the models based on the variables used in the risk scores. Our study is an attempt for internal validation of the ACC/AHA risk score for prediction of incident HF which may have led to overestimation of its performance. To assess the transferability of predictive models, they need to be externally validated to make it possible for them to be used in clinical settings [5, 6]. Also, because of unavailability of albumin, we were not able to include it in the Health ABC model. In addition, we did not have data on HF subtypes at the time of diagnosis. Inaccessibility of data on subtypes of HF is also a limitation of population-based cohort studies, like the ARIC and the health ABC studies. Moreover, our results might not be generalizable to younger individuals and other ethnicities.

## Conclusion

The model based on traditional risk factors included in the ACC/AHA model had an acceptable performance, comparable to more sophisticated models, for predicting 10-year HF among men and women from the general population. Our results therefore advocate use of this model for HF primary prevention. Addition of NT-proBNP to the ACC/AHA model leads to modest improvement in model performance, in particular among women. However, the clinical relevance of adding this biomarker for correct risk reclassification is limited.

## Supplementary Information

**Additional file 1: Supplementary Table 1**. Hazard ratios for incident HF in the ACC/AHA model, ARIC model, Health ABC model and ACC/AHA + NT-proBNP model in men and women. **Supplementary Table 2**. Overall goodness-of-fit for the ACC/AHA model, ARIC model, Health ABC model and ACC + NT-proBNP model in men and women. **Supplementary Table 3**. Risk reclassification for the ACC/AHA model after adding NT-proBNP stratified by event status. **Supplementary Table 4**. Fine and Gray’s subdistribution hazard ratios for incident HF and mortality in the ACC/AHA model, ARIC model, Health ABC model and ACC/AHA + NT-proBNP model in men and women. **Supplementary Figure 1**. Calibration plots for the observed and predicted risk based on the ACC/AHA model, ARIC model, Health ABC model and ACC + NT-proBNP model in men and women. **Supplementary Figure 2**. Risk reclassification for the ACC/AHA model after adding NT-proBNP stratified by event status.

## Data Availability

The analyzed datasets during the current study are not publicly available due to legal and ethical restraints. Sharing of individual participant data was not included in the informed consent of the study, and there is potential risk of revealing participants’ identities as it is not possible to completely anonymize the data. However, data are available from the corresponding author on reasonable request.
