# Supplementary material for: Implications of the ACC/AHA risk score for prediction of heart failure: the Rotterdam Study
Source: BMC Med. 2021 Feb 16;19:43. doi: 10.1186/s12916-021-01916-7 (PMC7885616; doi:10.1186/s12916-021-01916-7)
Supplement: Supplementary file 1 — Additional file 1: Supplementary Table 1. Hazard ratios for incident HF in the ACC/AHA model, ARIC model, Health ABC model and ACC/AHA + NT-proBNP model in men and women. Supplementary Table 2. Overall goodness-of-fit for the ACC/AHA model, ARIC model, Health ABC model and ACC + NT-proBNP model in men and women. Supplementary Table 3. Risk reclassification for the ACC/AHA model after adding NT-proBNP stratified by event status. Supplementary Table 4. Fine and Gray’s subdistribution hazard ratios for incident HF and mortality in the ACC/AHA model, ARIC model, Health ABC model and ACC/AHA + NT-proBNP model in men and women. Supplementary Figure 1. Calibration plots for the observed and predicted risk based on the ACC/AHA model, ARIC model, Health ABC model and ACC + NT-proBNP model in men and women. Supplementary Figure 2. Risk reclassification for the ACC/AHA model after adding NT-proBNP stratified by event status. [file 12916_2021_1916_MOESM1_ESM.docx]

Supplementary tables

Supplementary Table 1. Hazard ratios for incident HF in the ACC/AHA model, ARIC model, Health ABC model and ACC/AHA+NT-proBNP model in men and women

Supplementary Table 2. Overall goodness-of-fit for the ACC/AHA model, ARIC model, Health ABC model and ACC/AHA+NT-proBNP model in men and women

Supplementary Table 3. Risk reclassification for the ACC/AHA model after adding NT-proBNP stratified by event status

Supplementary Table 4. Fine and Gray's subdistribution hazard ratios for incident HF and mortality in the ACC/AHA model, ARIC model, Health ABC model and ACC/AHA+NT-proBNP model in men and women

Supplementary Figure 1. Calibration plots for the observed and predicted risk based on the ACC/AHA model, ARIC model, Health ABC model and ACC/AHA+NT-proBNP model in men and women

Supplementary Figure 2. Risk reclassification for the ACC/AHA model after adding NT-proBNP stratified by event status

Supplementary Table 1. Hazard ratios for incident HF in the ACC/AHA model, ARIC model, Health ABC model and ACC/AHA+NT-proBNP model in men and women

|  | **Men** | **Women** |
| --- | --- | --- |
| ACC/AHA model | **HR (95%CI)** | **HR (95%CI)** |
| Age | 1.10 (1.09 to 1.12) | 1.1. (1.09 to 1.11) |
| Total cholesterol | 0.97 (0.88 to 1.08) | 0.95 (0.86 to 1.05) |
| HDL cholesterol | 0.56 (0.40 to 0.77) | 0.83 (0.66 to 1.05) |
| Systolic blood pressure | 1.01 (1.01 to 1.02) | 1.01 (1.00 to 1.01) |
| Antihypertensive use | 2.87 (0.77 to 10.6) | 4.78 (1.41 to 16.2) |
| Smoking(current) | 1.44 (1.15 to 1.80) | 1.12 (0.85 to 1.46) |
| Prevalent diabetes | 1.31 (1.03 to 1.67) | 1.15 (0.89 to 1.49) |
| Systolic blood pressure: Antihypertensive use | 1.00 (0.99 to 1.01) | 0.99 (0.99 to 1.00) |
| ARIC model | **HR (95%CI)** | **HR (95%CI)** |
| Age | 1.07 (1.05 to 1.09) | 1.07 (1.06 to 1.09) |
| Log (NT-proBNP) | 1.72 (1.57 to 1.90) | 2.03 (1.83 to 2.26) |
| Heart rate | 1.01 (1.00 to 1.01) | 2.33 (0.70 to 7.70) |
| BMI | 1.09 (1.05 to 1.12) | 1.00 (1.00 to 1.01) |
| Systolic blood pressure | 1.01 (1.00 to 1.01) | 1.06 (1.03 to 1.08) |
| Antihypertensive use | 1.27 (0.35 to 4.67) | 1.00 (1.00 to 1.01) |
| Smoking(current) | 2.1 (1.39 to 3.15) | 1.24 (0.94 to 1.65) |
| Smoking(past) | 1.47 (1.01 to 2.15) | 1.22 (1.00 to 1.48) |
| Prevalent diabetes | 1.21 (0.95 to 1.54) | 1.25 (0.96 to 1.62) |
| Prevalent CHD | 1.33 (1.05 to 1.68) | 1.51 (1.06 to 2.15) |
| Systolic blood pressure: Antihypertensive use | 1.00 (0.99 to 1.01) | 1.00 (0.99 to 1.00) |
| Health ABC model | **HR (95%CI)** | **HR (95%CI)** |
| Age | 1.10 (1.08 to 1.11) | 1.11 (1.09 to 1.12) |
| Systolic blood pressure | 1.01 (1.01 to 1.01) | 1.01 (1.00 to 1.01) |
| Smoking(current) | 2.14 (1.42 to 3.22) | 1.33 (1.00 to 1.76) |
| Smoking(past) | 1.64 (1.13 to 2.39) | 1.38 (1.14 to 1.67) |
| Prevalent CHD | 2.09 (1.68 to 2.60) | 2.45 (1.73 to 3.47) |
| Heart rate | 1.00 (1.00 to 1.01) | 1.00 (0.99 to 1.01) |
| LVH | 2.10 (1.58 to 2.78) | 2.21 (1.58 to 3.10) |
| Creatinine | 1.00 (1.00 to 1.01) | 1.00 (1.00 to 1.01) |
| Glucose | 1.09 (1.04 to 1.14) | 1.10 (1.04 to 1.15) |
| ACC/AHA+NT-proBNP model | **HR (95%CI)** | **HR (95%CI)** |
| Age | 1.07 (1.05 to 1.08) | 1.07 (1.06 to 1.08) |
| Log (NT-proBNP) | 1.73 (1.58 to 1.90) | 2.02 (1.82 to 2.25) |
| Total cholesterol | 1.03 (0.93 to 1.14) | 1.01 (0.92 to 1.11) |
| HDL cholesterol | 0.52 (0.37 to 0.71) | 0.78 (0.62 to 0.98) |
| Systolic blood pressure | 1.01 (1.00 to 1.01) | 1.00 (1.00 to 1.01) |
| Antihypertensive use | 1.97 (0.56 to 6.98) | 3.20 (0.98 to 10.5) |
| Smoking(current) | 1.34 (1.07 to 1.67) | 1.07 (0.82 to 1.39) |
| Prevalent diabetes | 1.35 (1.06 to 1.72) | 1.35 (1.04 to 1.75) |
| Systolic blood pressure: Antihypertensive use | 1.07 (1.05 to 1.08) | 0.99 (0.99 to 1.00) |

BMI; body mass index, CHD; coronary heart disease, Diabetes; diabetes mellitus, HDL; high-density lipoprotein, LVH; left ventricular hypertrophy, HR; hazard ratio, CI; confidence interval

Supplementary Table 2. Overall goodness-of-fit for the ACC/AHA model, ARIC model, Health ABC model and ACC+NT-proBNP model in men and women

|  | | **Men** | | **Women** |  |
| --- | --- | --- | --- | --- | --- |
| Models | **AIC** | | | | **degrees of freedom** |
| ACC/AHA | | 6213.38 | 7503.19 | | 8 |
| ARIC | | 5953.16 | 6908.03 | | 11 |
| Health ABC | | 5651.17 | 6656.15 | | 9 |
| ACC/AHA + NT-proBNP | | 5970.96 | 7179.38 | | 9 |

AIC: Akaike information criterion

Supplementary Table 3. Risk reclassification for the ACC/AHA model after adding NT-proBNP stratified by event status

|  | Percent  event,%^*^ | | Event  NRI (95% CI),%^†^ | Percent  non-event,%^*^ | | Non-event  NRI (95% CI),%^†^ |
| --- | --- | --- | --- | --- | --- | --- |
|  | **Up** | **Down** |  | **Up** | **Down** |  |
| Men | 1.40 | 25.18 | -23.8 (-19.2 to -28.4) | 0.22 | 57.08 | 57.9 (54.8 to 61.0) |
| Women | 5.52 | 33.13 | -27.6 (-30.7 to -24.5) | 2.25 | 55.08 | 52.8 (50.3 to 55.5) |

^†^ NRI (95% CI) for reclassification of events and non-events after adding NT-proBNP to the ACC/AHA model. Event NRI was calculates as: (number of events reclassified up minus number of events reclassified down) / total number of events . Non-event NRI was calculates as: (number of non-events reclassified up minus number of non-events reclassified down) / total number of non-events

^*^ Percentages of persons with or without an event who moved to a higher (up) or lower risk (down) category after extension of the ACC/AHA model with NT-proBNP.

Supplementary Table 4. Fine and Gray's subdistribution hazard ratios for incident HF and mortality in the ACC/AHA model, ARIC model, Health ABC model and ACC/AHA+NT-proBNP model in men and women

|  | Heart failure | | Mortality | |
| --- | --- | --- | --- | --- |
|  | Men | Women | Men | Women |
| ACC/AHA model | **HR (95%CI)** | **HR (95%CI)** | **HR (95%CI)** | **HR (95%CI)** |
| Age | 1.06 (1.04 to 1.07) | 1.05 (1.04 to 1.06) | 1.09 (1.08 to 1.10) | 1.10 (1.10 to 1.11) |
| Total cholesterol | 0.99 (0.89 to 1.10) | 0.99 (0.89 to 1.08) | 0.95 (0.69 to 1.02) | 1.00 (0.93 to 1.07) |
| HDL cholesterol | 0.59 (0.44 to 0.80) | 0.91 (0.72 to 1.13) | 0.97 (0.80 to 1.19) | 0.83 (0.71 to 0.97) |
| Systolic blood pressure | 1.01 (1.00 to 1.03) | 1.02 (1.00 to 1.03) | 1.01 (1.00 to 1.02) | 1.00 (0.99 to 1.01) |
| Antihypertensive use | 2.34 (0.64 to 8.59) | 4.54 (1.36 to 15.2) | 1.89 (0.75 to 4.80) | 1.10 (0.44 to 2.71) |
| Smoking(current) | 1.14 (1.10 to 1.42) | 0.95 (0.73 to 1.25) | 1.56 (1.36 to 1.79) | 1.79 (1.55 to 2.07) |
| Prevalent diabetes | 1.19 (0.94 to 1.53) | 1.02 (0.78 to 1.33) | 1.18 (0.99 to 1.40) | 1.22 (1.01 to 1.46) |
| Systolic blood pressure: Antihypertensive use | 1.00 (0.99 to 1.01) | 0.99 (0.99 to 1.00) | 1.00 (0.99 to 1.00) | 1.00 (1.00 to 1.01) |
| ARIC model | **HR (95%CI)** | **HR (95%CI)** | **HR (95%CI)** | **HR (95%CI)** |
| Age | 1.03 (1.02 to 1.05) | 1.03 (1.02 to 1.05) | 1.08 (1.07 to 1.09) | 1.09 (1.08 to 1.10) |
| Log (NT-proBNP) | 1.49 (1.34 to 1.66) | 1.63 (1.45 to 1.83) | 1.09 (1.02 to 1.17) | 1.10 (1.02 to 1.18) |
| Heart rate | 1.00 (1.00 to 1.01) | 1.00 (0.99 to 1.01) | 1.01 (1.00 to 1.01) | 1.01 (1.00 to 1.01) |
| BMI | 1.09 (1.06 to 1.13) | 1.06 (1.04 to 1.08) | 0.97 (0.95 to 0.99) | 0.98 (0.96 to 0.99) |
| Systolic blood pressure | 1.01 (0.99 to 1.02) | 1.01 (1.00 to 1.02) | 1.01 (1.00 to 1.02) | 1.00 (1.00 to 1.01) |
| Antihypertensive use | 1.08 (0.29 to 4.10) | 2.51 (0.75 to 8.38) | 1.89 (1.74 to 4.82) | 1.34 (0.57 to 3.14) |
| Smoking(current) | 1.74 (1.17 to 2.58) | 1.07 (0.79 to 1.43) | 1.58 (1.25 to 1.99) | 1.77 (1.51 to 2.07) |
| Smoking(past) | 1.55 (1.08 to 2.22) | 1.26 (1.03 to 1.54) | 1.07 (0.86 to 1.32) | 1.00 (0.88 to 1.14) |
| Prevalent diabetes | 1.07 (0.83 to 1.38) | 1.00 (0.76 to 1.32) | 1.20 (1.01 to 1.43) | 1.34 (0.12 to 1.60) |
| Prevalent CHD | 1.41 (1.10 to 1.80) | 1.40 (0.93 to 2.10) | 0.95 (0.79 to 1.16) | 1.79 (0.52 to 1.20) |
| Systolic blood pressure: Antihypertensive use | 1.00 (0.99 to 1.01) | 1.00 (0.99 to 1.00) | 1.00 (0.99 to 1.00) | 1.00 (0.99 to 1.00) |
| Health ABC model | **HR (95%CI)** | **HR (95%CI)** | **HR (95%CI)** | **HR (95%CI)** |
| Age | 1.05 (1.04 to 1.06) | 1.10 (1.51 to 1.07) | 1.09 (1.08 to 1.10) | 1.10 (1.10 to 1.11) |
| Systolic blood pressure | 1.01 (1.01 to 1.02) | 1.01 (1.00 to 1.01) | 1.00 (1.00 to 1.00) | 1.00 (1.00 to 1.00) |
| Smoking(current) | 1.73 (1.17 to 2.57) | 1.12 (0.84 to 1.49) | 1.66 (0.32 to 2.09) | 1.82 (1.55 to 2.13) |
| Smoking(past) | 1.65 (1.43 to 2.38) | 1.38 (1.14 to 1.68) | 1.07 (0.86 to 1.32) | 1.01 (0.88 to 1.15) |
| Prevalent CHD | 1.99 (1.59 to 2.51) | 2.18 (1.49 to 3.18) | 1.00 (0.84 to 1.20) | 0.91 (0.61 to 1.35) |
| Heart rate | 1.00 (0.99 to 1.01) | 1.00 (0.99 to 1.00) | 1.01 (1.00 to 1.01) | 1.01 (1.00 to 1.01) |
| LVH | 2.08 (1.53 to 2.82) | 1.88 (1.31 to 2.71) | 0.80 (0.61 to 1.04) | 0.87 (0.63 to 1.20) |
| Creatinine | 1.00 (1.00 to 1.01) | 1.00 (0.99 to 1.00) | 1.00 (1.00 to 1.00) | 1.00 (1.00 to 1.01) |
| Glucose | 1.06 (1.01 to 1.11) | 1.07 (1.02 to 1.13) | 1.04 (1.00 to 1.08) | 1.02 (0.98 to 1.06) |
| ACC/AHA+NT-proBNP model | **HR (95%CI)** | **HR (95%CI)** | **HR (95%CI)** | **HR (95%CI)** |
| Age | 1.03 (1.01 to 1.04) | 1.03 (1.02 to 1.04) | 1.10 (1.08 to 1.10) | 1.10 (1.08 to 1.11) |
| Log (NT-proBNP) | 1.49 (1.35 to 1.65) | 1.63 (1.45 to 1.82) | 1.09 (1.02 to 1.17) | 1.10 (1.02 to 1.18) |
| Total cholesterol | 1.04 (0.94 to 1.15) | 1.04 (0.95 to 1.14) | 0.96 (0.90 to 0.03) | 1.01 (0.94 to 1.08) |
| HDL cholesterol | 0.57 (0.42 to 0.77) | 0.86 (0.69 to 1.08) | 0.97 (0.79 to 1.18) | 0.82 (0.71 to 0.96) |
| Systolic blood pressure | 1.01 (1.00 to 1.02) | 1.01 (1.00 to 1.03) | 1.01 (1.00 to 1.01) | 1.00 (0.99 to 1.01) |
| Antihypertensive use | 1.90 (0.52 to 6.93) | 3.45 (1.10 to 11.2) | 1.72 (0.68 to 4.37) | 1.08 (0.44 to 2.65) |
| Smoking(current) | 1.04 (1.83 to 1.31) | 0.89 (0.68 to 1.17) | 1.53 (1.33 to 1.76) | 1.78 (1.54 to 2.06) |
| Prevalent diabetes | 1.21 (0.94 to 1.55) | 1.10 (0.84 to 1.44) | 1.16 (0.97 to 1.39) | 1.23 (1.02 to 1.49) |
| Systolic blood pressure: Antihypertensive use | 1.00 (0.99 to 1.01) | 0.99 (0.99 to 1.00) | 1.00 (0.99 to 1.00) | 1.00 (0.99 to 1.01) |

BMI; body mass index, CHD; coronary heart disease, Diabetes; diabetes mellitus, HDL; high-density lipoprotein, LVH; left ventricular hypertrophy, HR; hazard ratio, CI; confidence interval

Supplementary Figure 1. Calibration plots for the observed and predicted risk based on the ACC/AHA model, ARIC model, Health ABC model and ACC+NT-proBNP model in men and women





Supplementary Figure 2. Risk reclassification for the ACC/AHA model after adding NT-proBNP stratified by event status


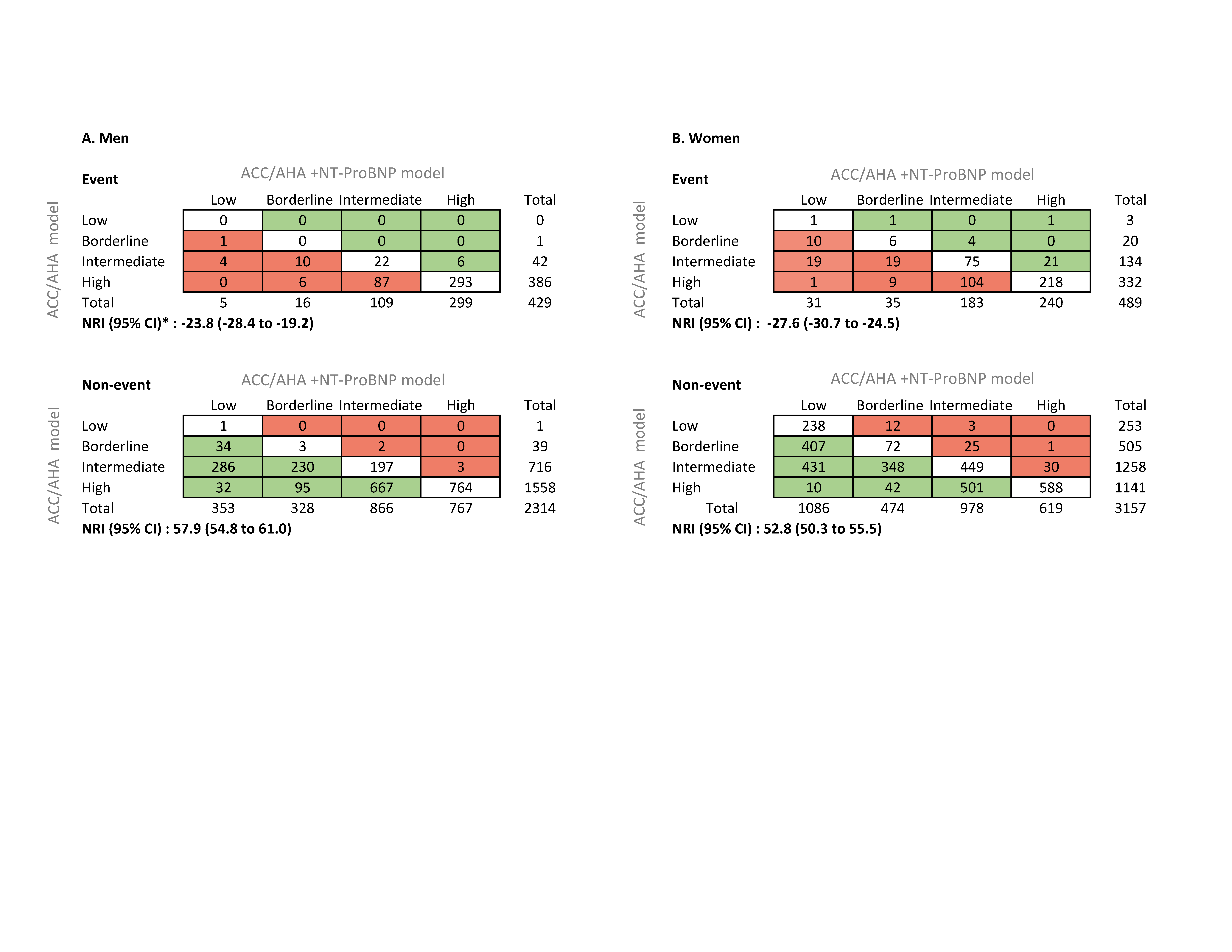


† Persons with or without an event (Event and non-event, respectively) who moved to a higher or lower risk category after extension of the ACC/AHA model with NT-proBNP. Favorable direction of reclassification is depicted in green (downward for non-events and upward for events) and unfavorable movement (downward for events and upward for non-events) is depicted in red.

*Event NRI was calculates as: (number of events reclassified up minus number of events reclassified down) / total number of events . Non-event NRI was calculates as: (number of non-events reclassified up minus number of non-events reclassified down) / total number of non-events
